# Severe Generalized Epidemic Myalgia in an Adult due to Human Parechovirus Type 3: A Case Report

**DOI:** 10.7759/cureus.30587

**Published:** 2022-10-22

**Authors:** Sakue Masuda, Kazuya Koizumi, Morihiko Sato, Haruki Uojima, Karen Kimura, Takashi Nishino, Chikamasa Ichita, Akiko Sasaki, Makomo Makazu, Masahiro Kobayashi, Jun Kubota, Chihiro Sumida

**Affiliations:** 1 Gastroenterology Medicine Center, Shonan Kamakura General Hospital, Kamakura, JPN; 2 Department of prevention and control of infection, Shonan Kamakura General Hospital, Kamakura, JPN; 3 Department of Gastroenterology, Internal Medicine, Kitasato University School of Medicine, Sagamihara, JPN

**Keywords:** adults, parechovirus type 3, aperture disorder, orchiodynia, epidemic myalgia, human parechovirus type 3, parechovirus

## Abstract

Parechovirus A type 3 (PeVA3) is most commonly transmitted to adults from children. Although PeVA3 infection is rarely diagnosed, as the symptoms are generally mild and self-limiting, this infection has been associated with epidemic myalgia in Japan. The patient, a 37-year-old man, presented with severe generalized myalgia, inability to open his mouth, and orchitis, which resolved over a period of 10 days. All members of his family were thought to have been infected with PeVA3 during a visit to an amusement park. Although the source of infection and inability to open his mouth are atypical, the acute generalized muscle symptoms made us suspect epidemic myalgia and enabled us to make a diagnosis of PeVA3 infection.

## Introduction

Parechoviruses (PeVs), genus *Parechovirus*, and family *Picornaviridae* are non-enveloped viruses with a positive single-stranded RNA genome. The genus Parechovirus comprises six species, Parechovirus A-F, with Parechovirus A (PeVA), formerly known as human parechovirus, consisting of 19 types, PeVA1-19. PeVAs have been identified worldwide, with PeVA1 and PeVA3 being the most frequently identified, followed by PeVA6 and PeVA4 [1].

PeVA3 was first reported in 2004 and has subsequently been found to cause cold-like symptoms, diarrhea, or severe infections such as meningitis, myositis, and sepsis-like disease in neonates [2,3]. The number of cases of PeVA1 and PeVA3 infection diagnosed, and the number of institutions reporting infections, have increased in Japan since 2008, and PeVA3 outbreaks have been reported every 2-3 years [1]. This may be because public health laboratories run by local governments in Japan introduced reverse transcription polymerase chain reaction (RT-PCR) testing for PeVs at that time [1].

Adult cases of epidemic myalgia associated with PeVA3 (PeVA3-M) were first reported in Japan in 2012 [4]. Notably, to date, all reported cases of epidemic myalgia due to PeVA3 in adults have occurred in Japan, despite the ubiquity of PeVA3 infection in other countries in Europe and Asia, and the USA [5]. Epidemic myalgia in association with PeVA3 infection presents as myalgia and weakness in the arms and legs; however, PeVA3 is rarely diagnosed in adults because severe cases are rare, and laboratory diagnostic tests are not routinely available. This report describes an adult with PeVA3 infection, though to have been acquired during a visit to an amusement park in Chiba Prefecture, Japan. The patient presented with severe generalized myalgia, inability to open his mouth, and orchitis.

## Case presentation

A previously healthy 37-year-old man presented to the outpatient department of our hospital in October 2019. His chief complaint was myalgia in the whole right arm that had appeared two days earlier. The myalgia had subsequently spread to the distal left upper arm the previous day, with the appearance of a mild sore throat and stomatitis. His symptoms worsened, and he visited the outpatient department of our hospital because he developed a fever of 40°C and was unable to open the lid of a plastic bottle owing to muscle weakness.

The patient had not traveled outside the country in the previous 10 years but had visited an amusement park in Chiba Prefecture in Japan with his family (wife, daughter, and two sons) one week before the onset of his illness. Around the time of the onset of his symptoms, his seven-year-old daughter developed ankle arthritis and a rash on her leg, and his two-year-old son developed a rash on his trunk and lower legs.

On physical examination during the first outpatient visit, the patient had mucocutaneous eruptions on the tongue and oral mucosa. His deep tendon reflexes were normal and there were no signs of joint tenderness, but his scrotum was tender. He was fully conscious, with no speech disturbance, skin eruptions, conjunctivitis, or cervical lymphadenopathy. His grip strength was 32 kg on the right and 28 kg on the left. Manual muscle testing revealed weakness in the arms (score: 4). His white blood cell count was 6,500/µL (normal range: 3,000-9,700/µL). Blood biochemistry revealed elevated levels of C-reactive protein (CRP) (0.86 mg/dL; normal: < 0.1 mg/dL), creatine phosphokinase (CK) (1,525 U/L; normal range: 52-192 U/L), and CK-MB (26.0 U/L; normal range: 0-25 U/L). Liver function tests showed elevated levels of aspartate aminotransferase (81 IU/L; normal range: 12-35 U/L), alanine aminotransferase (101 IU/L; normal range: 6-40 U/L), γ-glutamyl transferase (119 IU/L; normal range: 0-48 U/L), and lactate dehydrogenase (312 IU/L; normal range: 119-229 U/L), with a normal alkaline phosphatase level (273 U/L; normal range: 115-359 U/L). Electrolyte levels were within normal limits (Table 1).

**Table 1:** Laboratory findings at the first outpatient visit AST, aspartate aminotransferase; ALT, alanine aminotransferase; γGTP, γ-glutamyl transferase; ALP, alkaline phosphatase; CRP, C-reactive protein; BUN, blood urea nitrogen; Cr, creatinine; CK, creatine phosphokinase; LDH, lactate dehydrogenase

Laboratory test	Normal range	Result	Unit
Total bilirubin	0.1-1.2	0.7	mg/dL
AST	12-35	81	U/L
ALT	6-40	101	U/L
γ-GTP	0-48	119	U/L
ALP	115-359	273	U/L
CRP	<0.5	0.86	mg/dL
BUN	7.4-19.5	15.2	mg/dL
Cr	0.5-1.2	0.86	mg/dL
CK	52-192	1525	U/L
CK-MB	0-25	26	U/L
LDH	119-229	312	U/L
Leukocyte count	3000-9700	6500	/uL
Hemoglobin	13.1-17.6	15.2	g/dL
Platelet count	12.4-30.5	26.6	10^4^/uL

The initial differential diagnosis included epidemic myalgia, periodic paralysis, myasthenia gravis, Guillain-Barré syndrome, polymyositis, drug-induced myalgia, thyroid dysfunction, adult-onset Still disease, fibromyalgia, and chronic fatigue syndrome.

**Figure 1:**
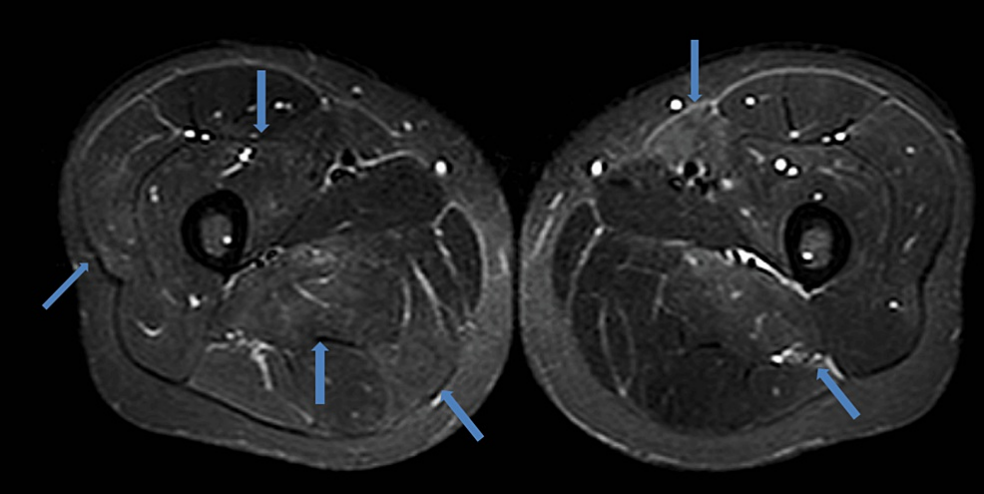
Magnetic resonance imaging short TI inversion recovery of the thighs (transverse view) Magnetic resonance imaging short TI inversion recovery of the thighs (transverse view) showing a weak, heterogeneous high signal in the fascia and the muscles of the abductor, adductor, quadriceps, and hamstring muscles of both thighs (blue arrows).

**Figure 2:**
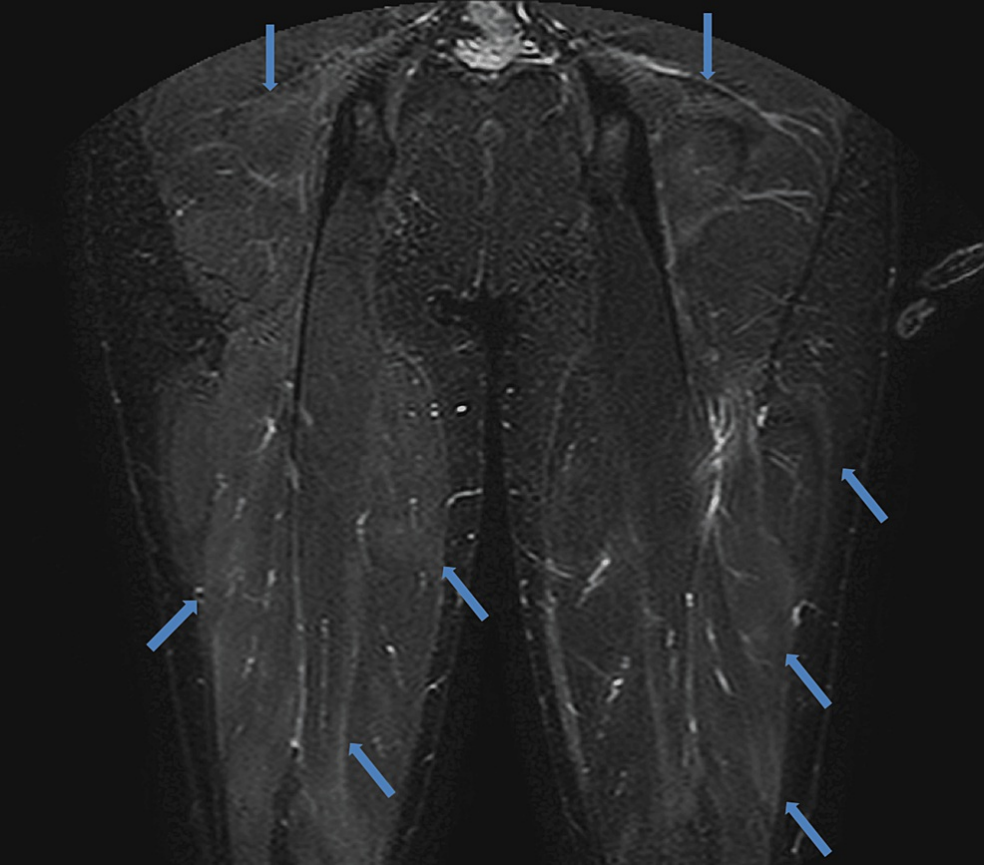
Magnetic resonance imaging short TI inversion recovery of the thighs (anteroposterior view) Magnetic resonance imaging short TI inversion recovery of the thighs (anteroposterior view) showing weak, heterogeneous high signal in the fascia and the muscles of gluteus, abductor, adductor, quadriceps, and hamstring muscles of both thighs (blue arrows).

Nerve conduction studies of the right arm showed normal motor and sensory conduction, normal latency, and no decrease in the frequency of F waves. Magnetic resonance imaging short TI inversion recovery (MRI-STIR) of the buttocks and thighs revealed mild, heterogeneous high signals in the fascia and the gluteus, abductor, adductor, quadriceps, and hamstring muscles (Figures 1, 2). An antigen-based rapid diagnostic test for influenza A and B was negative; however, because of the intensity of symptoms, oseltamivir was started at 150 mg/day and acetaminophen at 2,000 mg/day for suspected influenza.

The next day, the symptoms worsened, with myalgia extending to the masseter and sternocleidomastoid muscles. He had difficulty writing and opening his mouth. Two days after the outpatient visit, his body temperature returned to normal, but he developed myalgia in the quadriceps muscles, difficulty walking and descending stairs, and weakness in the legs. The myalgia and weakness in both arms worsened. Three days after the outpatient visit, the myalgia spread to the rectus abdominis muscle, and the patient was unable to turn over in bed.

We suspected epidemic myalgia based on the patient's acute intensity symptoms, onset after visiting an amusement park, and simultaneous onset of similar conditions in family members. To check the possibility of enterovirus infection, his serum was tested for antibodies to coxsackievirus (types B1, B2, B3, B4, B5, and B6) and echovirus (types 21, 22, 24, 25, and 30) at the initial visit, using the neutralization test method, but none of the titers were elevated. In addition, samples from the patient and his son were tested for PeVA3 using an RT-PCR test. The PeVA3 RT-PCR test results of the patient’s initial blood, pharyngeal swab, and stool sample, and his son’s stool and urine samples were positive. This confirmed the diagnosis of severe epidemic myalgia caused by PeVA3 infection (Table 2).

**Table 2:** Antibodies findings to virus

Test for antibodies to virus	Result
Echovirus types	
21	4
22	512
24	<4
25	4
30	4
Coxsackievirus types	
B1	<4
B2	16
B3	64
B4	128
B5	<4
B6	<4
Parechovirus A type 3	
Blood	Positive
Stool	Positive
Urine	Negative
Pharyngeal swab	Positive

Four days after the outpatient visit, the symptoms decreased and he was able to walk. Five days after the outpatient visit, oseltamivir and acetaminophen were terminated. One week after the outpatient visit, all symptoms had almost disappeared. The serum antibody tests for coxsackievirus and echovirus were not repeated two weeks later because the diagnosis of PeVA3 infection had been confirmed.

## Discussion

On the first outpatient visit, we suspected the influenza based on the patient's acute myalgia, a family cluster, and that the symptoms started approximately a week after the family had visited an amusement park. Influenza virus and enterovirus infection are more common than PeVA3 infection as causes of myalgia and myositis [5-7]. Epidemic pleurisy (also called Bornholm's disease), commonly caused by coxsackievirus type B and characterized by fever and paroxysmal convulsions of the chest and abdominal muscles [6,7]. However, myalgia and muscle weakness predominantly in the limbs, especially preventing the patient from standing, walking, and writing, are key signs of suspected PeVA3-M [1]. In addition, orchiodynia is frequently found in the acute phase of PeVA3-M, as in this case. A study by Mizuta et al. [1] found that among adults, epidemic myalgia associated with PeVA3 (PeVA3-M) occurs predominantly (87.7%) in the age group of 30-49 years. The sex ratio (male-to-female) was 39:10 in adults. The most prominent clinical features were myalgia (100%) and weakness (84.3-100%). In men, fever was present in 80.4-82.4%, respiratory symptoms in 52.9%, and testicular symptoms in 28.6-35.9%, but fever tended to be absent in women. Mizuta et al. [4] also reported 22 adult PeVA3-M cases in which severe myalgia occurred mainly in the proximal portions of the arms and legs and was generally not materialized. As no specific treatment is available for PeVA3 infection, preventive measures such as careful and frequent hand-washing and environmental cleansing are important [8-10]. Patients with PeVA3-M generally deteriorate rapidly within two to three days of onset, but recover without sequelae within one to two weeks with conservative treatment [1]. To date, no serious sequelae have been reported. Elevated CPK levels normalize within a few days, and the MRI signal intensity improves approximately 10 days after admission, suggesting a good prognosis [4,11]. In most cases, the changes in the leukocyte count and the CRP level are minimal, and the CK level may be only slightly elevated, despite severe myalgia and weakness [5]. Our patient had some atypical features, namely, myalgia of the masseter muscle and difficulty opening the mouth, and no difference between the right and left legs, but the distal symptoms were particularly severe in the right arm.

PeVA3 is thought to circulate among infants in day care facilities and in the home. However, most associated cases of myalgia occur in adults, and the infection is thought to be transmitted from infants to adults [12]. Previous reports have shown that most adults with epidemic myalgia due to PeVA3 infection are aged between 30 and 49 years, the age group responsible for child care [4,11,13,14]. PeVA3 seropositivity decreases with age [15]. This suggests that adults with inadequate neutralizing antibody titers may develop PeVA3 infection through occasional contact with infected children and may develop epidemic myalgia as a complication. However, in the present case, the onset of symptoms occurred simultaneously in the patient and his son and daughter; therefore, it appears that the patient did not acquire the infection from his children, and the most likely source is the crowded amusement park in Chiba Prefecture in Japan, which the family visited a week prior to the symptom onset. To our knowledge, amusement parks have not been reported as a source of infection previously.

The reason why adult epidemic myalgia due to PeVA3 has only been reported in Japan is unknown. Possible reasons include racial variation, differences in the clinical testing system, and recognition of this disease by general physicians (i.e., non-pediatricians) [5]. Mizuta et al. [1] pointed out that the condition cannot be diagnosed unless the physician has a sufficient knowledge of PeVA3-M to consider it as a possible diagnosis. PeVA3-M is difficult to diagnose as it tends to occur sporadically. Individuals do not always seek medical attention because the myalgia varies from mild to severe and recovers in a short period of time. Even when individuals with PeVA3-M seek medical care, they visit a variety of departments, including neurology, internal medicine, pediatrics, urology, and orthopedics, and many specialists are not familiar with the condition and thus may fail to make an accurate diagnosis. Therefore, PeVA3-M is likely to have been reported by a limited number of physicians with an academic interest in infectious disease working in a limited geographic area where laboratory diagnosis is possible.

## Conclusions

In summary, when generalized muscle pain and weakness are present in Japanese adults under 50 years of age, and there are no abnormal neurological findings, PeVA3 should be suspected. It is important to note that PeVA3-M may be present even in cases of myalgia of the masseter muscle, difficulty opening the mouth, or left-right differences in symptoms, as in the present case, and that PeVA3-M may be present even if the CK level is not markedly elevated. In addition, when taking a medical history, it is important to be aware that transmission can occur in crowded places, as in this case, and not only from contact with children. Individuals with PeVA3-M do not require treatment, as they generally recover without sequelae within one to two weeks with conservative treatment. However, it is important to recognize PeVA3 because an accurate diagnosis can prevent misdiagnosis and incorrect treatment.
